# Short-Term Functional Trajectories After Surgery in Older Adults: National Patterns of Loss and Recovery in 436,471 Patients

**DOI:** 10.1097/AS9.0000000000000654

**Published:** 2026-04-02

**Authors:** Dany Y. Matar, Charbel G. Saad, Anahita Nimbalkar, Sue-Yan Lai, Thomas Schaschinger, Sarah Friedrich, Jasmin Rühl, Samuel Knoedler, Leonard Knoedler, Steffen Koerdt, Carsten Rendenbach, Leila Harhaus-Waehner, Max Heiland, Dennis P. Orgill, Gabriel Hundeshagen, Adriana C. Panayi

**Affiliations:** From the * Division of Plastic Surgery, Department of Surgery, Brigham and Women’s Hospital, Harvard Medical School, Boston, MA; † Department of Plastic and Reconstructive Surgery, Johns Hopkins University School of Medicine, Baltimore, MD; ‡ Department of Acute Medicine, Royal Free Hospital, London, UK; § BG Klinik Ludwigshafen, Department of Hand, Plastic, and Reconstructive Surgery, Burn Center at Heidelberg University, Ludwigshafen, Germany; ‖ Department of Mathematical Statistics and Artificial Intelligence in Medicine, University of Augsburg, Augsburg, Germany; ¶ Department of Oral and Maxillofacial Surgery, Charité – Universitätsmedizin Berlin, corporate member of Freie Universität Berlin, Humboldt-Universität zu Berlin, Berlin, Germany; # Department of Hand, Replantation, and Microsurgery BG Klinikum Unfallkrankenhaus Berlin and Chair of Hand, Replantation, and Microsurgery at the Charité University Medicine Berlin, Berlin, Germany; ** Department of Cranio-Maxillofacial and Oral Surgery, University Hospital Zurich, University of Zurich, Zurich, Switzerland.

**Keywords:** big data, loss of independence, outcomes, quality improvement, surgery

## Abstract

**Objective:**

To characterize short-term functional trajectories and identify risk factors for loss and recovery of independence among older surgical patients at a national level.

**Background:**

As the surgical population ages, frailty increasingly influences outcomes beyond mortality. Short-term postoperative functional independence is a key patient-centered outcome with long-term implications, yet national data across surgical specialties remain limited.

**Methods:**

We conducted a retrospective cohort study using the 2022–2024 American College of Surgeons National Surgical Quality Improvement Program database, including all patients aged ≥75 years. Patients were categorized by functional trajectory from admission to 30 days postdischarge: maintenance of independence, loss of independence (LOI), maintenance of dependence, or gain of independence (GOI). Multivariable logistic regression identified independent factors associated with LOI among baseline-independent patients, and with GOI among baseline-dependent patients. Prespecified subgroup analyses assessed effect modification by dementia, age, frailty (mFI-5 score), surgical specialty, surgical approach, urgency, and setting.

**Results:**

Among 436,471 patients, 28.2% of baseline-independent adults experienced LOI, while 5.6% of baseline-dependent patients experienced GOI. LOI rates increased stepwise with age and frailty and were strongly associated with frailty, recent falls, preoperative sepsis, higher American Society of Anesthesiologists class, urgent/emergent surgery, inpatient setting, and open surgical approaches. LOI was associated with markedly higher postoperative mortality, prolonged hospitalization, delirium, sepsis, ventilator dependence, and nonhome discharge. Dementia was independently associated with LOI risk and inversely with GOI risk across nearly all subgroups.

**Conclusions:**

Short-term LOI is common among adults aged ≥75 years and represents a powerful marker of adverse postoperative complications. Dementia and perioperative acuity are dominant associated factors of short-term functional trajectory, while short-term recovery of independence is rare. Findings suggest that short-term functional outcomes should be incorporated into preoperative risk stratification, shared decision-making, and perioperative care pathways for older adults.

## INTRODUCTION

The demand for surgical care among older adults continues to rise as populations age, with over 1 in 5 individuals over 75 undergoing a surgical procedure each year.^1,2^ This demographic shift has led to a steady increase in the median age of surgical patients over the past decade.^3^ Aging is closely linked to frailty, and extensive evidence shows that both aging and frailty diminish physiologic reserve and impair wound healing, cardiovascular stability, metabolic response, and immune function.^4–8^

Despite these risks, the primary outcomes emphasized in most large-scale surgical outcomes research remain traditional endpoints such as morbidity and mortality. While these are critical measures, they do not fully reflect the priorities of older surgical patients. Quality of life and functional status are often valued more than survival alone; 70% to 90% of older adults report that they would decline interventions likely to result in severe cognitive or functional decline, even if such treatments extend life.^9^ Functional independence, defined as the ability to perform daily activities, maintain mobility, and avoid institutional care, is increasingly recognized as a central patient-centered outcome in perioperative decision-making.

Previous studies have examined postoperative functional decline and identified important risk factors, including patient demographics, frailty measures, and comorbidities.^10–15^ However, existing research remains limited by several factors. First, most studies have focused on single-center or specialty-specific cohorts with limited generalizability across surgical populations.^10–13^ Second, prior National Surgical Quality Improvement Program (NSQIP)-based analyses, including 1 study by Zhang et al,^14^ which evaluated 30-day functional decline in approximately 2000 older surgical patients, have been constrained by sample size, limiting the ability to stratify risk by frailty, cognitive status, procedure type, surgical approach, operative urgency, or care setting.

Understanding how these factors interact to influence short-term functional trajectories is critical for several reasons. For older adults, the return to preoperative independence can be slow and uncertain, often requiring up to 6 months of recovery.^10^ Extended recovery times, in turn, heighten the risk of complications, rehospitalizations, and functional decline.^11,16–18^ Identifying patients at risk for loss of independence (LOI) at the time of discharge and within the first 30 days after surgery is therefore critical for setting realistic expectations, guiding perioperative planning, and informing decisions regarding postacute care needs.

We analyzed data from the American College of Surgeons NSQIP (ACS-NSQIP) from 2022 to 2024, which includes perioperative outcomes from over 700 hospitals and more than 400,000 older adults undergoing diverse surgical procedures. Building on prior work, we sought to characterize the patient-, procedure-, and system-level factors associated with short-term postoperative functional decline across a broad, contemporary, multispecialty surgical cohort to inform risk stratification and surgical decision-making.

## METHODS

### Data Source

The ACS-NSQIP is a nationally validated, risk-adjusted program that evaluates surgical care quality, collecting data from over 700 hospitals, mainly in the United States. It covers various surgical procedures and over 150 perioperative variables. Trained personnel input data from medical charts, ensuring reliability through peer reviews and audits. This study used 2022 to 2024 ACS-NSQIP data, as discharge functional status and surgical approach were not included before 2022. Ethical approval was granted by Brigham and Women’s Hospital (Protocol #: 2020P001675).

### Patient Selection

**FIGURE 1.**
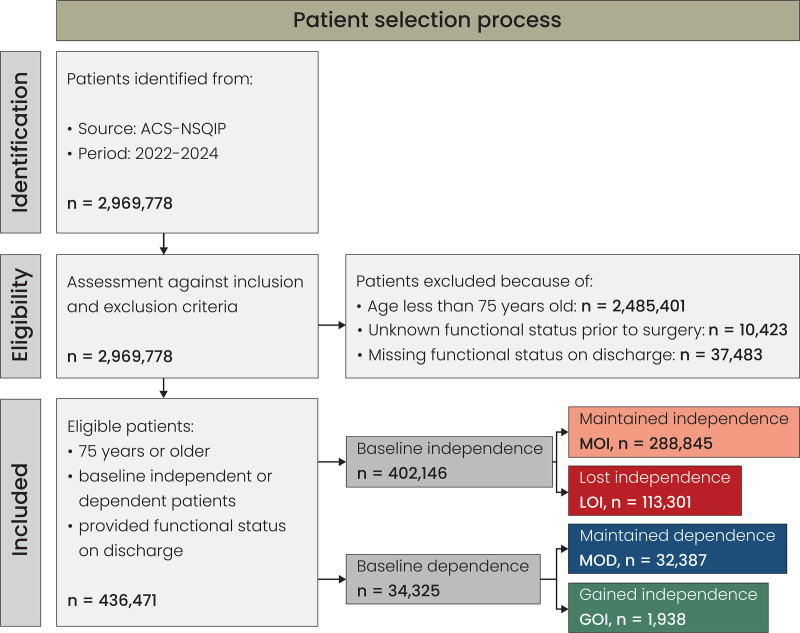
Patient selection process. Flow diagram depicting cohort identification from the ACS-NSQIP database (2022–2024), application of inclusion and exclusion criteria, and derivation of the final analytic sample of patients aged ≥75 years with documented functional status at discharge. Eligible patients were stratified by baseline functional status (independent vs dependent) and categorized by postoperative functional trajectories: MOI, LOI, MOD, or GOI, with corresponding sample sizes shown.

We identified all patients aged ≥75 years in the 2022 to 2024 ACS-NSQIP database. With rising life expectancy, this age threshold has been suggested as a more clinically relevant marker than conventional definitions of older age (such as 65 years) for identifying surgical patients who are at increased risk of postoperative complications and loss of functional independence.^19–21^ To reduce confounding, we only excluded cases with missing pre- or discharge functional status. The screening and selection process is shown in Figure 1.

### Variable Extraction

All variables reported by the ACS-NSQIP were extracted for all included patients. Preoperative variables included patient demographics, comorbidities, laboratory values, home support, and origin status (Table 1). Preoperative frailty was quantified using the modified frailty index-5 (mFI-5), calculated as the unweighted sum of 5 preoperative deficits (diabetes, chronic obstructive pulmonary disease, congestive heart failure, hypertension requiring medication, and partial or total functional dependence).

**TABLE 1.**
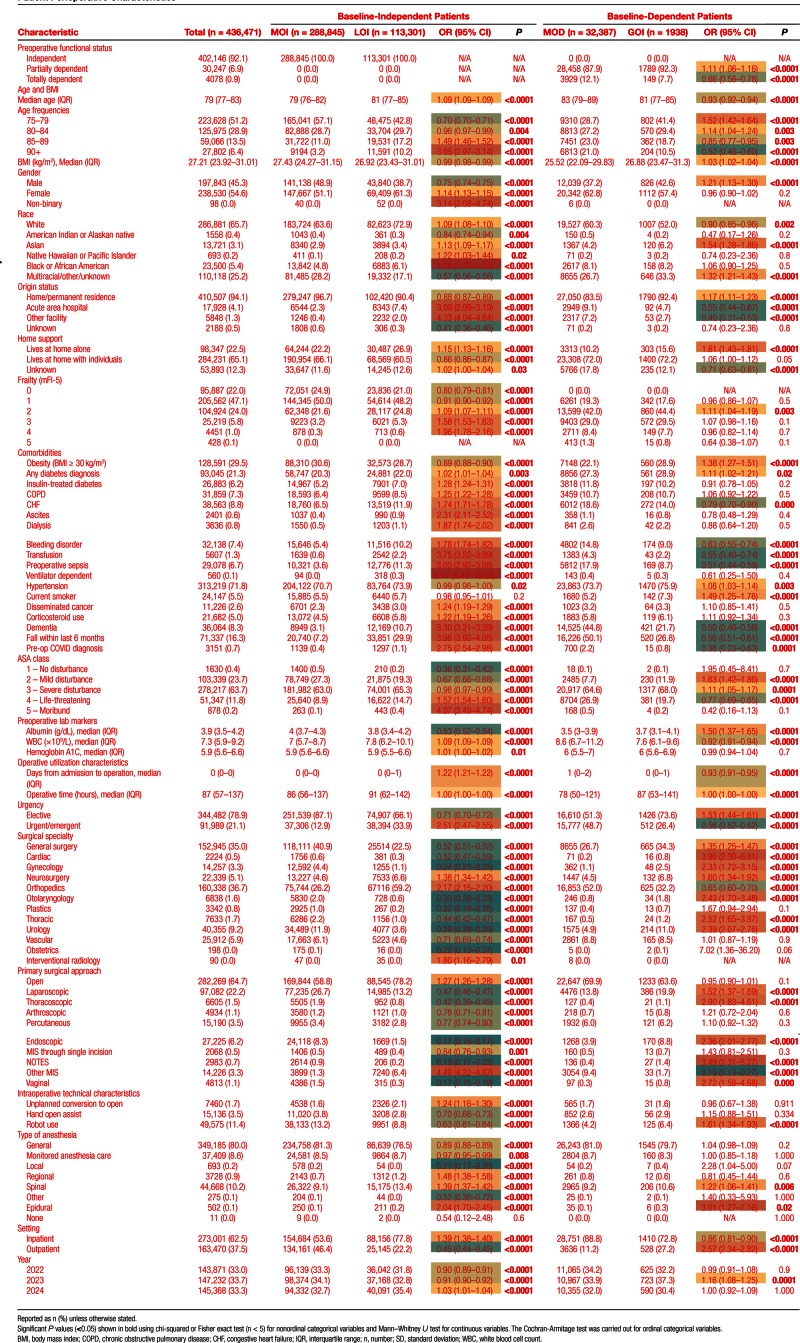
Patient Perioperative Characteristics

Surgical variables captured days from admission to operation, operative time, urgency, anesthesia type, surgical specialty, procedure setting, surgical approach, and year of surgery (Table 1).

Postoperative variables within 30 days included mortality, length of stay, days from operation to discharge, reoperation, end-of-life or withdrawal of care, readmission, surgical and medical complications, use of immunosuppressive or oxygen therapy, discharge destination, home discharge services, and postoperative functional status.

### Functional Status

Per the ACS-NSQIP definitions, functional status describes a patient’s ability to carry out basic activities of daily living (ADLs) during the 30 days preceding surgery and 30 days postdischarge. The highest level achieved is recorded within each time period. Functional status determination by NSQIP surgical clinical reviewers (SCRs) involves a comprehensive manual chart review of multiple documentation sources within 30 days before surgery, as the data are rarely explicitly stated in standardized form. Functional status is reported in 3 categories:

1. Independent: Performs all ADLs without human assistance.
2. Partially dependent: Requires some help from another person to complete ADLs.
3. Totally dependent: Needs complete assistance from another person for all ADLs.

### Statistical Analysis

Raw ACS-NSQIP data were analyzed and visualized using GraphPad Prism (Version 8.00, MacOS). Patients were categorized by functional status trajectory from admission to discharge into 4 groups: maintenance of independence (MOI), LOI, maintenance of dependence (MOD), and gain of independence (GOI). MOI included patients who were independent preoperatively and remained so upon discharge. LOI included patients who declined from independent to partially/totally dependent. MOD included patients who were partially/totally dependent preoperatively and remained so on discharge. GOI included those who improved from partially/totally dependent to independent.

Categorical variables were reported as counts and percentages and compared using Pearson’s χ^2^ or Fisher exact test (n < 5). Continuous variables were reported as means ± standard deviations (SD) or medians with interquartile ranges (IQR), assessed for normality with the D’Agostino–Pearson test, and compared using 2-sample *t* tests or Mann–Whitney tests. Given the large sample size, odds ratios (OR) with 95% confidence intervals (CI) were calculated to assess the magnitude and clinical relevance of associations.

Two multivariable logistic regression models identified independent associated factors of LOI and GOI, including all preoperative and intraoperative variables. All preoperative and intraoperative variables available in the ACS-NSQIP were extracted and tabulated for included patients (Table 1) and considered as covariates for multivariable analysis, with variables only excluded for the following reasons: preoperative functional status served as part of our outcome definition; diabetes, chronic obstructive pulmonary disease, congestive heart failure, and hypertension are components of mFI-5 and would introduce collinearity; race had high missingness (25.2% unknown/multiracial); ascites and ventilator dependence had very low prevalence (<1%); and laboratory values (albumin, white blood cell count, and HbA1c) had substantial missingness. All multivariable models reported adjusted ORs (aORs) with 95% CIs.

Prespecified subgroup analyses assessed effect modification of the association between preoperative dementia (primary exposure) and postoperative functional transitions. Separate multivariable models were fit within strata of age, mFI-5, American Society of Anesthesiologists (ASA) class, transfer status, operative urgency, surgical approach, specialty, home support, and care setting. LOI was modeled among patients independent at baseline, and GOI among those dependent at baseline. Models adjusted for all pre- and intraoperative covariates from the primary analysis. Adjusted risk ratios (aRRs) with 95% CIs compared patients with versus without dementia.

Unadjusted probabilities of LOI and GOI were calculated by stratifying patients by dementia status, operative urgency (elective vs urgent/emergent), and mFI-5 category. LOI was estimated among baseline-independent patients and GOI among baseline-dependent patients as the proportion experiencing each outcome within strata. GOI was not estimated for mFI-5 = 0 due to baseline dependence.

*P* values were Bonferroni-adjusted for multiple testing where applicable, and *P* < 0.05 was considered statistically significant for all analyses.

## RESULTS

We analyzed 436,471 older adults (≥75 years) from the 2022 to 2024 ACS-NSQIP dataset, of whom 288,845 (66.2%) had MOI, 113,301 (26.0%) experienced LOI, 32,387 (7.4%) had MOD, and 1938 (0.4%) had GOI (Fig. 1).

### Perioperative Characteristics Among Baseline-Independent Patients: Maintenance of Independence Versus Loss of Independence

LOI rose progressively with age, and patients aged ≥90 years were overrepresented in the LOI group compared with the MOI group (10.2% vs 3.2%), corresponding to a threefold higher odds of LOI (OR = 3.05; 95% CI = 2.97–3.14; *P* < 0.0001). Severe frailty was also strongly associated with mFI-5 = 4, associated with nearly doubled odds of LOI (OR = 1.96; 95% CI = 1.78–2.16; *P* < 0.0001).

Dementia was associated with a threefold increase in LOI compared with MOI (10.7% vs 3.1%; OR = 3.30; 95% CI = 3.21–3.39; *P* < 0.0001), and a recent fall was associated with nearly fourfold higher odds (29.9% vs 7.2%; OR = 3.98; 95% CI = 3.92–4.05; *P* < 0.0001). Additional high-risk factors included preoperative sepsis (11.3% vs 3.6%; OR = 3.00; 95% CI = 2.92–3.08; *P* < 0.0001), transfusion (2.2% vs 0.6%; OR = 3.75; 95% CI = 3.52–3.99; *P* < 0.0001), ventilator dependence (OR = 8.17; 95% CI = 6.49–10.28; *P* < 0.0001), while higher albumin was associated with lower odds of LOI (OR = 0.53 per unit; 95% CI = 0.52–0.54; *P* < 0.0001). Urgent/emergent surgery had more than doubled LOI odds (33.9% vs 12.9%; OR = 2.51; 95% CI = 2.47–2.55; *P* < 0.0001).

LOI was associated with substantially worse postoperative outcomes beyond expected increases in length of stay (median 4 vs 1 days; OR = 1.15/day; 95% CI = 1.15–1.15; *P* < 0.0001) (Supplemental Table 1, https://links.lww.com/AOSO/A582). Patients experiencing LOI had markedly higher mortality (1.9% vs 0.2%; OR = 7.36; 95% CI = 6.77–8.01; *P* < 0.0001) and end-of-life care or withdrawal (2.3% vs 0.2%; OR = 9.65; 95% CI = 8.85–10.51; *P* < 0.0001). Several complications showed particularly large associations with LOI, including ventilator dependence >48 hours (1.2% vs 0.1%; OR = 8.10; 95% CI = 7.25–9.04), septic shock (2.1% vs 0.3%; OR = 5.96; 95% CI = 5.53–6.42), delirium (8.7% vs 1.8%; OR = 4.63; 95% CI = 4.48–4.79), reintubation (0.9% vs 0.2%; OR = 4.23; 95% CI = 3.83–4.67), and pneumonia (3.1% vs 0.7%; OR = 3.90; 95% CI = 3.70–4.12) (all *P* < 0.0001).

### Perioperative Characteristics Among Baseline-Dependent Patients: Maintenance of Dependence Versus Gain of Independence

GOI generally showed inverse associations with LOI (Table 1). Patients aged 75 to 79 years comprised a large proportion of GOI patients (41.4%), while those aged ≥90 years had nearly 50% lower odds of GOI (10.5% vs 21.0% MOD; OR = 0.52; 95% CI = 0.46–0.60; *P* < 0.0001).

Dementia patients had 50% reduced odds of GOI compared with MOD (21.7% vs 44.8%; OR = 0.50; 95% CI = 0.46–0.56; *P* < 0.0001), and a recent fall had a similar effect (26.8% vs 50.1%; OR = 0.56; 95% CI = 0.51–0.61; *P* < 0.0001). Higher albumin was associated with increased GOI (median 3.7 vs 3.5 g/dL; OR = 1.50, 95% CI = 1.37–1.65; *P* < 0.0001). Outpatient surgery was associated with more than doubled GOI odds relative to inpatient procedures (27.2% vs 11.2%; OR = 2.57; 95% CI = 2.34–2.82; *P* < 0.0001), and elective surgery similarly was associated with increased GOI odds (73.6% vs 51.3%; OR = 1.53; 95% CI = 1.44–1.61; *P* < 0.0001).

GOI was associated with shorter hospitalization (median LOS 3 vs 6 days; OR = 0.93; 95% CI = 0.93–0.94; *P* < 0.0001) and markedly lower mortality (1.5% vs 4.9%; OR = 0.33; 95% CI = 0.23–0.47; *P* < 0.0001), as well as reduced end-of-life care (1.3% vs 6.1%; OR = 0.23; 95% CI = 0.16–0.34; *P* < 0.0001) (Supplemental Table 1, https://links.lww.com/AOSO/A582). GOI was negatively associated with ventilator dependence >48 hours (OR = 0.29; 95% CI = 0.14–0.61), delirium (OR = 0.38; 95% CI = 0.32–0.46), renal failure (OR = 0.53; 95% CI = 0.39–0.71), sepsis (OR = 0.55; 95% CI = 0.39–0.78), and postoperative COVID-19 (OR = 0.44; 95% CI = 0.29–0.68) (all *P* ≤ 0.001).

### Patient Origin and Discharge by Functional Trajectory

**FIGURE 2.**
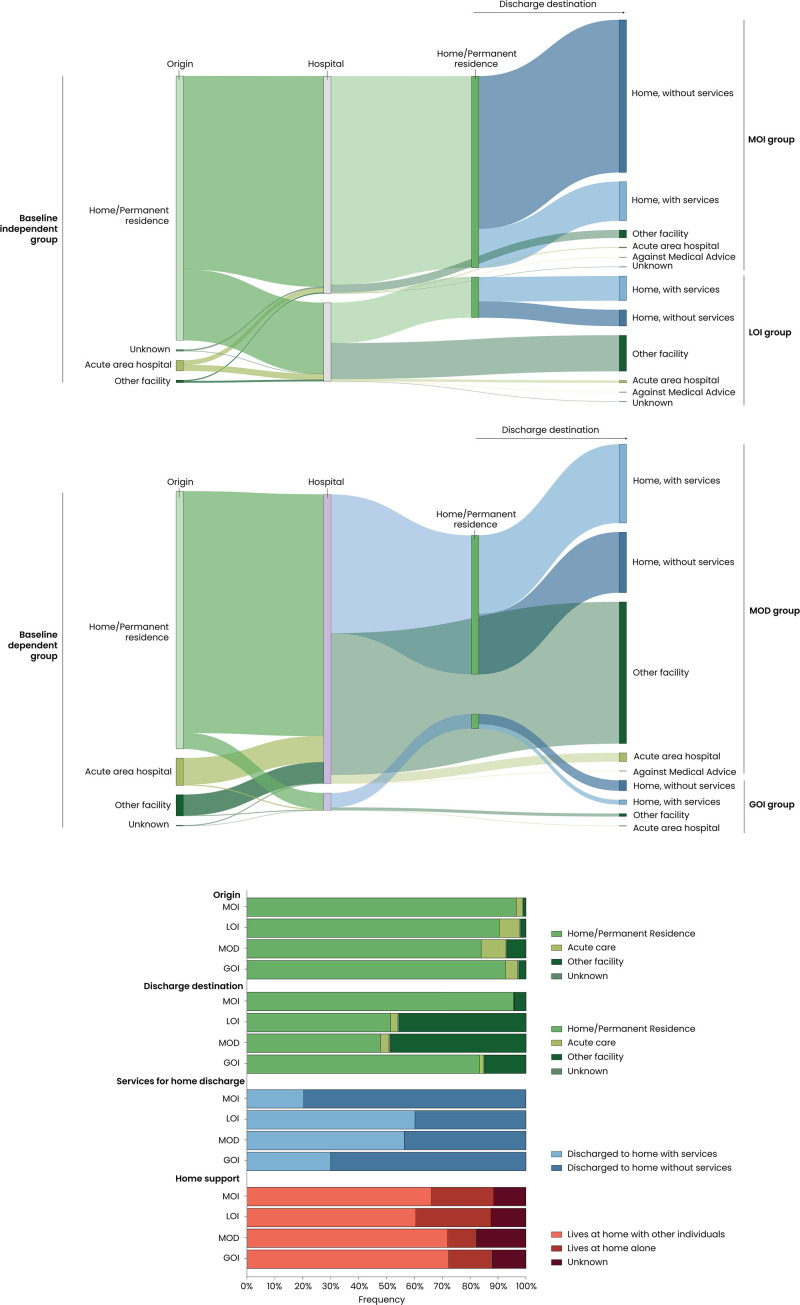
Sankey visualization of patient origin, discharge destination, and postdischarge support. Sankey diagrams depict divergent perioperative care trajectories among patients stratified by baseline functional status, comparing baseline-independent patients who MOI with those who experienced LOI, and baseline-dependent patients who MOD with those who GOI.

Patient trajectories from admission to discharge are illustrated in Figure 2.

Nonhome origin status was strongly associated with LOI, including transfers from other facilities (OR = 4.33; 95% CI = 4.04–4.64; *P* < 0.0001) and acute care hospitals (OR = 3.09; 95% CI = 2.99–3.19; *P* < 0.0001). Patients experiencing LOI were substantially less likely to be discharged home without services (20.4% vs 76.2%; OR = 0.25; 95% CI = 0.24–0.25; *P* < 0.0001) and far more likely to require discharge to another facility (45.7% vs 3.8%; OR = 11.52; 95% CI = 11.28–11.76; *P* < 0.0001) or transfer to acute care (2.7% vs 0.4%; OR = 7.06; 95% CI = 6.58–7.57; *P* < 0.0001). In contrast, patients achieving GOI were more likely to return home overall (83.5% vs 48.0%; OR = 1.86; 95% CI = 1.77–1.96; *P* < 0.0001), particularly without services (58.6% vs 20.9%; OR = 3.00; 95% CI = 2.82–3.20; *P* < 0.0001), and were significantly less likely to require facility discharge (OR = 0.33; 95% CI = 0.29–0.37) or acute care transfer (OR = 0.31; 95% CI = 0.19–0.50) (both *P* < 0.0001).

### Confounder-Adjusted Multivariable Analyses

After multivariable adjustment, several factors demonstrated large and independent associations with postoperative LOI (Table 2). Advanced age showed a steep, dose-dependent association, with patients aged ≥90 years experiencing nearly threefold higher adjusted odds of LOI (aOR = 2.68; 95% CI = 2.57–2.78) compared with those aged 75 to 79 years. Severe frailty was associated with substantial adjusted odds, particularly at mFI-5 ≥3 (aOR = 1.75–2.10). Preoperative sepsis (aOR = 2.29; 95% CI = 2.21–2.38), dementia (aOR = 2.29; 95% CI = 2.21–2.38), recent falls (aOR = 1.89; 95% CI = 1.85–1.94), and perioperative transfusion (aOR = 1.71; 95% CI = 1.58–1.84) were among the strongest independently associated factors. Higher physiologic acuity, reflected by ASA class IV–V, was associated with particularly pronounced adjusted odds (ASA V: aOR = 5.38; 95% CI = 4.15–6.97). Urgent/emergent surgery was independently associated with increased LOI odds (aOR = 1.72; 95% CI = 1.68–1.77), as were inpatient procedures (aOR = 1.65; 95% CI = 1.62–1.68) and open surgical approaches (aOR = 1.55; 95% CI = 1.49–1.62).

**TABLE 2.**
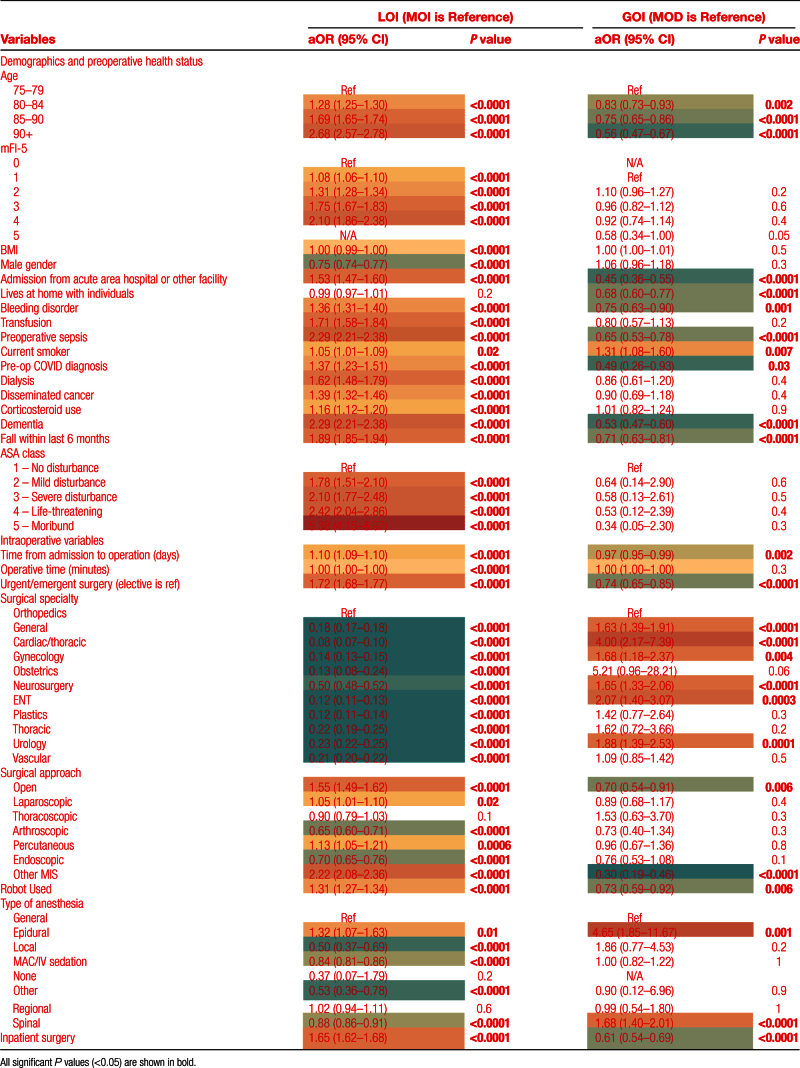
Multivariable Logistic Regression Models Examining Loss of Independence Among Patients Who Were Functionally Independent at Baseline, and GOI Among Those Who Were Functionally Dependent at Baseline, Adjusting for Preoperative and Intraoperative Covariates

Few variables demonstrated large independent protective or promotive associations for GOI. Dementia was the most prominent negatively associated covariate, associated with a lower adjusted odds of postoperative functional recovery by nearly half (aOR = 0.53; 95% CI = 0.47–0.60). Care transitions and acuity-related factors were independently associated with limited recovery, including admission from an acute care facility (aOR = 0.45; 95% CI = 0.36–0.55) and inpatient surgery (aOR = 0.61; 95% CI = 0.54–0.69). Certain anesthesia modalities demonstrated unexpectedly large independent associations with recovery: epidural anesthesia was associated with substantially higher adjusted odds of GOI (aOR = 4.65; 95% CI = 1.85–11.67), and spinal anesthesia was also strongly favorable (aOR = 1.68; 95% CI = 1.40–2.01), independent of case urgency and comorbidity burden.

### Confounder-Adjusted Subgroup Analyses

**FIGURE 3.**
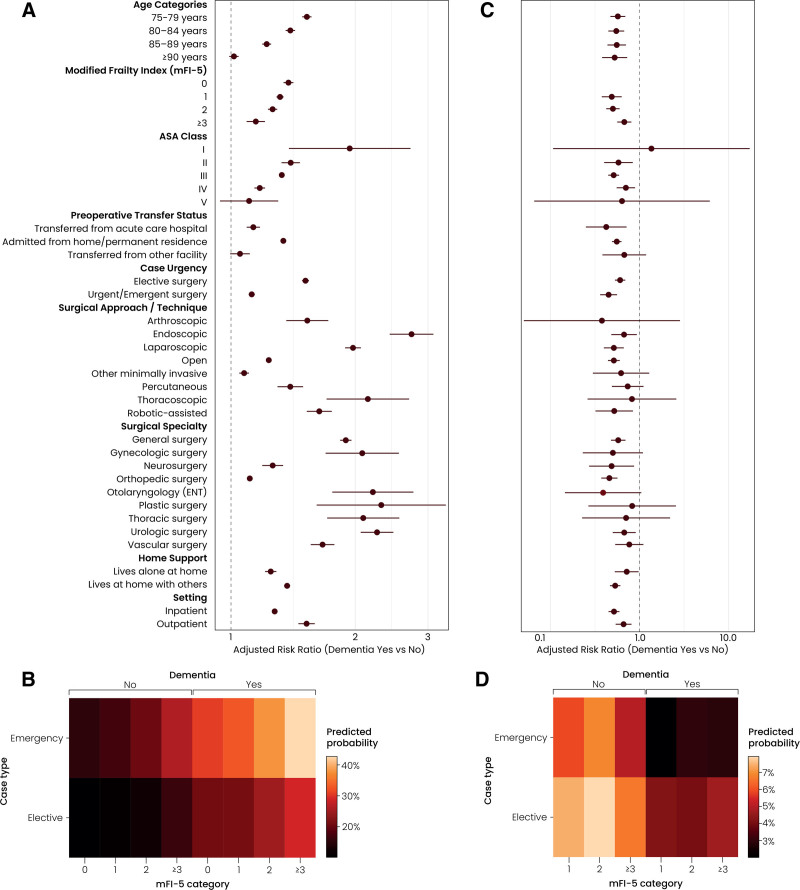
Subgroup analyses of LOI and GOI. Forest plots display multivariable-adjusted odds ratios for LOI among patients who were functionally independent at baseline and for GOI among those who were functionally dependent at baseline. Heat maps show model-predicted absolute risks stratified by modified Frailty Index-5 (mFI-5), dementia status, and case type.

Across nearly all prespecified subgroups, preoperative dementia was independently associated with a higher risk of postoperative LOI and a substantially lower risk of GOI compared with nondemented patients (Fig. 3 and Supplemental Tables 2 and 3, https://links.lww.com/AOSO/A582).

The excess LOI risk associated with dementia was consistent across age, frailty, ASA class, operative urgency, surgical approach, specialty, home support, and care setting, with aRRs generally ranging from ~1.1 to >2.0. The magnitude of association attenuated at the extremes of physiologic vulnerability (eg, age ≥90 years and ASA class 5), where estimates were smaller and, in some strata, not statistically significant (Fig. 3A and Supplemental Table 2, https://links.lww.com/AOSO/A582). In contrast, dementia was uniformly associated with markedly reduced GOI across most strata (aRRs typically ~0.4–0.7) (Fig. 3C and Supplemental Table 2, https://links.lww.com/AOSO/A582).

Absolute LOI risk increased monotonically with higher frailty, urgent/emergent surgery, and dementia (Figure 3B, Supplementary Table 3 https://links.lww.com/AOSO/A582). Among patients without dementia, LOI risk ranged from 10.1% (elective, mFI-5=0) to 26.1% (urgent/emergent, mFI-5≥3), whereas patients with dementia experienced substantially higher LOI risk across comparable strata, ranging from 21.0% to 42.7%. GOI probabilities were uniformly low and consistently lower among patients with dementia across all frailty and urgency categories (generally ~2–5% with dementia vs ~5–8% without dementia) (Figure 3D, Supplementary Table 3 https://links.lww.com/AOSO/A582).

## DISCUSSION

LOI is a critical yet often overlooked surgical outcome that impacts a patient’s ability to perform daily activities. This study is the first to detail short-term LOI rates across a large, multispecialty cohort of 436,471 older surgical patients. In this multi-institutional retrospective analysis, we found a 26.0% LOI rate. Several factors were linked to higher LOI, offering new insights into the complex factors associated with LOI in older adults.

Although LOI within 30 days of surgery does not necessarily indicate permanent disability, evidence shows it is a strong early predictor of long-term outcomes, including persistent functional decline, institutionalization, and mortality.^15,22–29^ Functional status at discharge strongly predicts 1-year outcomes; for instance, hip fracture patients with early declines in ADLs have a two- to fourfold increased risk of death or ongoing dependency.^27,29–33^ In our cohort, orthopedic and neurosurgical patients had some of the highest LOI rates, likely reflecting postoperative mobility limitations and neurologic deficits. Acute declines in these patients, who often require prolonged rehabilitation, carry significant prognostic weight.^32,33^ Importantly, the NSQIP end-of-life/withdrawal-of-care variable captures anticipated prognosis and postoperative goals of care rather than functional status; therefore, although patients may be classified as functionally independent at discharge while still carrying this designation, the higher prevalence of end-of-life/withdrawal-of-care status among patients who developed LOI in this study further reflects greater underlying illness severity and vulnerability to postoperative functional deterioration.

Discharge disposition in this study likely reflects the clinical consequences of postoperative functional status rather than an independent effect. Patients who remained independent were appropriately discharged home, whereas those experiencing LOI more often required facility-based care or home services, consistent with the support needs associated with functional decline. Discharge location may therefore function as a downstream marker of recovery at the time of discharge. The small proportion of functionally independent patients sent to facilities likely had care needs unrelated to functional status. In line with prior literature linking early postoperative functional decline to subsequent dependence and institutionalization,^26,31,34,35^ patients with LOI were more frequently discharged to institutional settings, identifying a subgroup at risk for adverse long-term functional trajectories. Although LOI was measured within 30 days, this early decline may represent a key inflection point in recovery and a proxy for longer-term outcomes.

Age is a well-established predictor of LOI, with older patients at significantly higher risk. In a study of 9972 general surgery patients, those ≥85 years had a fourfold higher risk of LOI than those aged 65 to 74.^16^ In our cohort, advancing age was strongly and progressively associated with LOI, with patients aged 80 to 84, 85 to 90, and ≥90 years demonstrating 28%, 69%, and 168% higher adjusted odds, respectively, compared with those aged 75 to 79, indicating a marked age-dependent risk gradient. However, chronological age alone may not fully capture vulnerability, as physiological age varies widely. Frailty, defined as an aging-related decline across multiple physiological systems that reduces resilience to stressors, offers a more precise measure of surgical risk.^8^

Frailty affects an estimated 10% to over 50% of surgical patients and is consistently associated with worse postoperative outcomes across specialties.^8,12,14,15,22–26^ Frail individuals are more likely to experience LOI and require discharge to nonhome settings.^13,21,26^ Frailty demonstrated a stepwise, dose–response association with LOI, with increasing mFI-5 scores conferring progressively higher adjusted odds, ranging from 8% higher odds at mFI-5 = 1 to more than double the odds at mFI-5 = 4 compared with nonfrail patients, reflecting diminished physiologic reserve and resilience to surgical stress.

Cognitive impairment, particularly dementia, was among the strongest associated factors of postoperative LOI, consistent with evidence that impaired cognition limits rehabilitation, accelerates functional decline, and increases mortality and rehospitalization risk.^36,37^ A history of falls within 6 months, an indicator of impaired mobility, balance, and underlying frailty, was also strongly associated with LOI, reinforcing prior findings that preoperative falls are associated with postoperative dependency and adverse outcomes.^27–29,32,37–40^ Collectively, these results highlight the central role of frailty-related factors in shaping postoperative functional trajectories.

Perioperative factors such as hospital length of stay, surgical approach, and operative duration emerged as key determinants of postoperative functional decline. Each additional preoperative inpatient day increased LOI risk by 10%, underscoring the impact of preoperative deconditioning. Although this per-day risk was lower than the 13% to 30% reported in high-risk surgical cohorts,^16,41^ prolonged inpatient stays of five or more days in those populations have been linked to more than 15-fold higher odds of LOI.^42^ This relationship likely reflects both physiologic deconditioning, most functional decline occurs within the first postoperative week and can persist for months,^10,11,16^and LOI itself, as delays in mobilizing discharge support may prolong hospitalization and increase nonhome discharge rates.^17^

Minimally invasive and other nonopen surgical approaches, including laparoscopic, arthroscopic, percutaneous, and endoscopic techniques, were associated with significantly lower adjusted odds of postoperative LOI compared with planned open procedures. These findings align with evidence that MIS reduces tissue trauma, dampens the inflammatory response, and accelerates recovery.^43,44^ Arthroscopic and endoscopic approaches demonstrated the strongest protective effects, likely due to their low morbidity and reduced physiologic stress, which may translate into faster mobilization, higher rates of home discharge, and improved long-term functional trajectories. In contrast, robotic assistance was associated with increased LOI risk, likely reflecting greater case complexity or longer operative times.^45–47^

## Limitations

The ACS-NSQIP’s voluntary participation may bias results toward larger academic centers, and data quality can vary by site. Its retrospective design introduces bias and lacks long-term follow-up (>30 days) on functional, mobility, and cognitive outcomes, though prior research shows minimal heterogeneity.^48^ ACS-NSQIP functional status relies on SCR abstraction from heterogeneous clinical documentation. When explicit ADL assessments are absent, SCRs infer status from diagnoses, living situation, equipment use, and physical examination findings. This may introduce misclassification bias, particularly for distinguishing partially from totally dependent patients. However, in our large multi-institutional cohort of 400,000+ patients across multiple specialties, surgical approaches, and procedures, functional status remained a robust associated factor of adverse postoperative outcomes, suggesting the classifications retain clinical relevance despite measurement imperfections. Reported associations are statistical, not causal. Finally, frailty, a key factor associated with poor outcomes, may mediate the effects of factors such as low BMI or dementia, but could not be assessed due to data limitations.

### Future Directions

These findings underscore the need for perioperative strategies that mitigate short-term functional decline and prioritize independence as a key patient-centered outcome. Early mobilization, targeted prehabilitation, and minimizing operative duration can reduce LOI risk, while multidisciplinary care, including early geriatric involvement, tailored discharge planning, and proactive identification of limited home support, further supports recovery.^49^ Future work should extend LOI assessment beyond discharge, incorporate standardized frailty, cognitive, and social support measures to enhance risk stratification, and evaluate enhanced recovery pathways and postdischarge rehabilitation. Such efforts will be critical to reducing the burden of LOI and preserving functional independence in the growing population of older surgical patients.

## CONCLUSIONS

In this large, multispecialty cohort, nearly 1 in 3 older surgical patients experienced LOI within 30 days, underscoring the need to prioritize functional outcomes alongside morbidity and mortality. Risk was shaped by age, cognitive status, prior falls, frailty, prolonged hospitalization, and surgical invasiveness. Although measured within 30 days after discharge, short-term LOI may mark patients at highest risk for lasting dependency who need targeted support. Optimizing surgical care for older adults requires equal focus on preserving independence and survival.

## ACKNOWLEDGMENTS

The authors would like to thank our Quality Program Manager, Jill Steinberg, MPH, RN, for her help with the ACS-NSQIP data acquisition. The American College of Surgeons National Surgical Quality Improvement Program and the hospitals participating in the ACS-NSQIP are the source of the data used herein; they have not verified and are not responsible for the statistical validity of the data analysis or the conclusions derived by the authors.

## Supplementary Material
