# Supplementary material for: Short-Term Functional Trajectories After Surgery in Older Adults: National Patterns of Loss and Recovery in 436,471 Patients
Source: Ann Surg Open. 2026 Apr 2;7(2):e654. doi: 10.1097/AS9.0000000000000654 (PMC13290161; doi:10.1097/AS9.0000000000000654)
Supplement: Supplementary file 1 [file as9-7-e654-s001.pdf]

Supplementary Table 1. **Peri- and postoperative outcomes**. Reported as n (%) unless otherwise stated. Significant p values ($p < 0.05$) shown in bold using chi-squared or Fisher's exact test for non-ordinal categorical variables and Student's t-test and Mann-Whitney U test for continuous variables. Cochran-Armitage test was carried out for ordinal categorical variables.

		Baseline-Independent Patients				Baseline-Dependent Patients			
**Characteristic**	**Total Patients (n=436471)**	**MOI (n =288845)**	**LOI (n =113301)**	**OR (95% CI)**	**p value**	**MOD (n =32387)**	**GOI (n =1938)**	**OR (95% CI)**	**p value**
**Healthcare utilization characteristics**									
Length of Stay (days) Median (IQR)	2 [1, 6]	1 [0, 3]	4 [2, 8]	1.15 (1.15-1.15)	**p < 0.0001**	6 [3, 11]	3 [1, 7]	0.93 (0.93-0.94)	**p < 0.0001**
Operation to Discharge (days), Median (IQR)	2 [1, 5]	1 [0, 3]	4 [1, 7]	1.17 (1.17-1.18)	**p < 0.0001**	5 [2, 8]	3 [1, 6]	0.92 (0.91-0.93)	**p < 0.0001**
**Complications**									
**Mortality**	4533 (1.0)	720 (0.2)	2193 (1.9)	7.36 (6.77-8.01)	**p < 0.0001**	1590 (4.9)	30 (1.5)	0.33 (0.23-0.47)	**p < 0.0001**
**Reoperation**	10824 (2.5)	5268 (1.8)	4240 (3.7)	1.95 (1.87-2.03)	**p < 0.0001**	1243 (3.8)	73 (3.8)	1.03 (0.81-1.31)	p = 0.9
**End of Life/Withdrawal of Care**	5275 (1.2)	653 (0.2)	2606 (2.3)	9.65 (8.85-10.51)	**p < 0.0001**	1990 (6.1)	26 (1.3)	0.23 (0.16-0.34)	**p < 0.0001**
**Readmission**	31749 (7.3)	16533 (5.7)	10828 (9.6)	1.58 (1.55-1.62)	**p < 0.0001**	4144 (12.8)	244 (12.6)	1.03 (0.91-1.18)	p = 0.6
**Unplanned Readmission**	31105 (7.1)	16070 (5.6)	10701 (9.4)	1.61 (1.57-1.65)	**p < 0.0001**	4096 (12.6)	238 (12.3)	1.02 (0.90-1.16)	p = 0.8
**Any Surgical Complication**	41637 (9.5)	17049 (5.9)	17968 (15.9)	2.56 (2.50-2.61)	**p < 0.0001**	6370 (19.7)	250 (12.9)	0.69 (0.61-0.78)	**p < 0.0001**
Superficial Incisional Infection	7044 (1.6)	4189 (1.5)	2116 (1.9)	1.22 (1.16-1.29)	**p < 0.0001**	699 (2.2)	40 (2.1)	1.00 (0.73-1.38)	p = 1.000
Deep Incisional Infection	1200 (0.3)	604 (0.2)	421 (0.4)	1.68 (1.49-1.91)	**p < 0.0001**	162 (0.5)	13 (0.7)	1.41 (0.80-2.48)	p = 0.3
Organ Space Infection	6856 (1.6)	3348 (1.2)	2786 (2.5)	2.01 (1.91-2.11)	**p < 0.0001**	680 (2.1)	42 (2.2)	1.08 (0.79-1.48)	p = 0.7
Dehiscence	1533 (0.4)	772 (0.3)	573 (0.5)	1.79 (1.61-2.00)	**p < 0.0001**	179 (0.6)	9 (0.5)	0.88 (0.45-1.72)	p = 0.8
Bleeding/Transfusion	27887 (6.4)	9126 (3.2)	13478 (11.9)	3.58 (3.49-3.68)	**p < 0.0001**	5110 (15.8)	173 (8.9)	0.59 (0.51-0.69)	**p < 0.0001**
**Any Medical Complication**	57844 (13.3)	22036 (7.6)	24862 (21.9)	2.74 (2.69-2.79)	**p < 0.0001**	10610 (32.8)	336 (17.3)	0.55 (0.50-0.62)	**p < 0.0001**
Pneumonia	7164 (1.6)	2153 (0.7)	3476 (3.1)	3.90 (3.70-4.12)	**p < 0.0001**	1484 (4.6)	51 (2.6)	0.60 (0.46-0.80)	**p < 0.0001**
Pulmonary embolism	2342 (0.5)	991 (0.3)	1071 (0.9)	2.61 (2.39-2.85)	**p < 0.0001**	267 (0.8)	13 (0.7)	0.85 (0.49-1.49)	p = 0.7
Reintubation	1982 (0.5)	602 (0.2)	1054 (0.9)	4.23 (3.83-4.67)	**p < 0.0001**	316 (1.0)	10 (0.5)	0.56 (0.30-1.04)	p = 0.08
Ventilator Dependence >48h	2215 (0.5)	409 (0.1)	1371 (1.2)	8.10 (7.25-9.04)	**p < 0.0001**	428 (1.3)	7 (0.4)	0.29 (0.14-0.61)	**p = 0.001**
Renal Insufficiency/Failure	9507 (2.2)	3315 (1.1)	4654 (4.1)	3.39 (3.25-3.55)	**p < 0.0001**	1493 (4.6)	45 (2.3)	0.53 (0.39-0.71)	**p < 0.0001**
Urinary Tract Infection	9995 (2.3)	5195 (1.8)	3414 (3.0)	1.59 (1.52-1.66)	**p < 0.0001**	1310 (4.0)	76 (3.9)	1.02 (0.81-1.28)	p = 0.9
Stroke	1443 (0.3)	466 (0.2)	751 (0.7)	3.89 (3.47-4.37)	**p < 0.0001**	219 (0.7)	7 (0.4)	0.56 (0.26-1.19)	p = 0.2
Cardiac Arrest	754 (0.2)	270 (0.1)	358 (0.3)	3.20 (2.73-3.75)	**p < 0.0001**	123 (0.4)	3 (0.2)	0.43 (0.14-1.35)	p = 0.2
Myocardial Infarction	3788 (0.9)	1543 (0.5)	1651 (1.5)	2.58 (2.41-2.77)	**p < 0.0001**	579 (1.8)	15 (0.8)	0.45 (0.27-0.76)	**p = 0.003**
Deep Vein Thrombosis	3376 (0.8)	1347 (0.5)	1582 (1.4)	2.84 (2.64-3.05)	**p < 0.0001**	431 (1.3)	16 (0.8)	0.65 (0.40-1.07)	p = 0.1
Sepsis	5988 (1.4)	2313 (0.8)	2595 (2.3)	2.71 (2.56-2.87)	**p < 0.0001**	1047 (3.2)	33 (1.7)	0.55 (0.39-0.78)	**p = 0.001**
Septic Shock	4104 (0.9)	952 (0.3)	2347 (2.1)	5.96 (5.53-6.42)	**p < 0.0001**	768 (2.4)	37 (1.9)	0.85 (0.61-1.18)	p = 0.4
Clostridium difficile infection	1834 (0.4)	879 (0.3)	713 (0.6)	1.96 (1.77-2.16)	**p < 0.0001**	227 (0.7)	15 (0.8)	1.16 (0.69-1.96)	p = 0.7
Delirium	20920 (4.8)	5159 (1.8)	9861 (8.7)	4.63 (4.48-4.79)	**p < 0.0001**	5773 (17.8)	127 (6.6)	0.38 (0.32-0.46)	**p < 0.0001**
Post-op COVID Diagnosis	5407 (1.2)	2235 (0.8)	2313 (2.0)	2.50 (2.36-2.65)	**p < 0.0001**	838 (2.6)	21 (1.1)	0.44 (0.29-0.68)	**p = 0.0001**
**Immunosuppressive Therapy**									
Steroids	11475 (2.6)	6588 (2.3)	3610 (3.2)	1.32 (1.27-1.38)	**p < 0.0001**	1200 (3.7)	77 (4.0)	1.13 (0.89-1.42)	p = 0.3
Anti-rejection/transplant	1766 (0.4)	1087 (0.4)	529 (0.5)	1.18 (1.06-1.30)	**p = 0.003**	142 (0.4)	8 (0.4)	0.99 (0.49-2.02)	p = 1.000
Synthetic DMARDs	2873 (0.7)	1855 (0.6)	816 (0.7)	1.06 (0.98-1.15)	p = 0.2	188 (0.6)	14 (0.7)	1.31 (0.76-2.25)	p = 0.4
Biologic DMARDs	6888 (1.6)	4158 (1.4)	2155 (1.9)	1.25 (1.19-1.32)	**p < 0.0001**	551 (1.7)	24 (1.2)	0.76 (0.51-1.15)	p = 0.2
Other	2158 (0.5)	1390 (0.5)	619 (0.5)	1.08 (0.98-1.18)	p = 0.1	141 (0.4)	8 (0.4)	1.00 (0.49-2.03)	p = 1.000
**Oxygen Therapy**	18856 (4.3)	5621 (1.9)	8651 (7.6)	3.73 (3.60-3.85)	**p < 0.0001**	4471 (13.8)	113 (5.8)	0.44 (0.37-0.53)	**p < 0.0001**
**Services for home discharge**									
Without services	251272 (57.6)	220219 (76.2)	23160 (20.4)	0.25 (0.24-0.25)	**p < 0.0001**	6758 (20.9)	1135 (58.6)	3.00 (2.82-3.20)	**p < 0.0001**
With services	100842 (23.1)	56309 (19.5)	35262 (31.1)	1.52 (1.50-1.54)	**p < 0.0001**	8788 (27.1)	483 (24.9)	0.96 (0.88-1.06)	p = 0.5
**Discharge destination**									
Home/Permanent Residence	352121 (80.7)	276534 (95.7)	58423 (51.6)	0.50 (0.49-0.50)	**p < 0.0001**	15546 (48.0)	1618 (83.5)	1.86 (1.77-1.96)	**p < 0.0001**
Acute care	5119 (1.2)	1055 (0.4)	3081 (2.7)	7.06 (6.58-7.57)	**p < 0.0001**	966 (3.0)	17 (0.9)	0.31 (0.19-0.50)	**p < 0.0001**
Other facility	78950 (18.1)	11066 (3.8)	51725 (45.7)	11.52 (11.28-11.76)	**p < 0.0001**	15856 (49.0)	303 (15.6)	0.33 (0.29-0.37)	**p < 0.0001**
Against Medical Advice	281 (0.1)	190 (0.1)	72 (0.1)	0.92 (0.70-1.20)	p = 0.6	19 (0.1)	0 (0.0)	N/A	N/A
**Discharge Functional Status**									
Independent	290783 (66.6)	288845 (100.0)	0 (0.0)	N/A	N/A	0 (0.0)	1938 (100.0)	N/A	N/A
Partially Dependent	134529 (30.8)	0 (0.0)	108553 (95.8)	N/A	N/A	25976 (80.2)	0 (0.0)	N/A	N/A
Totally Dependent	11159 (2.6)	0 (0.0)	4748 (4.2)	N/A	N/A	6411 (19.8)	0 (0.0)	N/A	N/A

Supplementary Table 2. Confounder-adjusted subgroup analyses of postoperative transitions in functional independence by dementia status. Preoperative dementia status was the primary exposure. Adjusted risk ratios (aRRs) with 95% confidence intervals (CIs) estimate the risk of loss of independence (LOI) among patients who were independent at baseline and the risk of gain of independence (GOI) among patients who were dependent at baseline, comparing patients with versus without dementia. Prespecified subgroup analyses were conducted across age category, frailty (mFI-5), ASA class, preoperative transfer status, operative urgency, surgical approach, surgical specialty, home support, and care setting. Multivariable models were adjusted for demographic, clinical, and procedural covariates. NA indicates insufficient events for stable estimation.

	**LOI (MOI is reference)**		**GOI (MOD is reference)**	
	**aRR (95% CI)**	**p-value**	**aRR (95% CI)**	**p-value**
**Age Category**				
75-79	1.53 (1.49, 1.57)	<0.001	0.57 (0.47, 0.69)	<0.001
80-84	1.39 (1.36, 1.43)	<0.001	0.55 (0.45, 0.67)	<0.001
85-89	1.22 (1.19, 1.25)	<0.001	0.56 (0.44, 0.71)	<0.001
90+	1.02 (0.99, 1.04)	0.221	0.53 (0.38, 0.73)	<0.001
**Modified Frailty Index (mFI-5)**				
0	1.38 (1.34, 1.42)	<0.001	NA	NA
1	1.31 (1.29, 1.34)	<0.001	0.49 (0.38, 0.63)	<0.001
2	1.26 (1.23, 1.29)	<0.001	0.50 (0.42, 0.60)	<0.001
3+	1.15 (1.09, 1.21)	<0.001	0.67 (0.56, 0.81)	<0.001
**ASA Class**				
1-No Disturb	1.94 (1.38, 2.72)	<0.001	1.36 (0.11, 17.23)	0.813
2-Mild Disturb	1.40 (1.33, 1.47)	<0.001	0.58 (0.40, 0.84)	0.004
3-Severe Disturb	1.33 (1.31, 1.35)	<0.001	0.51 (0.45, 0.59)	<0.001
4-Life Threat	1.17 (1.14, 1.21)	<0.001	0.70 (0.55, 0.89)	0.004
5-Moribund	1.11 (0.94, 1.30)	0.223	0.64 (0.07, 6.14)	0.696
**Preoperative Transfer Status**				
Acute care hospital	1.13 (1.09, 1.18)	<0.001	0.42 (0.25, 0.72)	0.001
Home/Permanent residence	1.34 (1.32, 1.36)	<0.001	0.56 (0.49, 0.63)	<0.001
Other facility	1.05 (1.00, 1.11)	0.073	0.67 (0.38, 1.19)	0.171
**Case Urgency**				
Elective	1.52 (1.49, 1.55)	<0.001	0.61 (0.53, 0.69)	<0.001
Urgent/Emergent	1.12 (1.10, 1.14)	<0.001	0.45 (0.36, 0.56)	<0.001
**Surgical Approach/Technique**				
Arthroscopic	1.53 (1.36, 1.72)	<0.001	0.38 (0.05, 2.84)	0.345
Endoscopic	2.74 (2.42, 3.09)	<0.001	0.67 (0.48, 0.93)	0.017
Laproscopic	1.97 (1.89, 2.06)	<0.001	0.52 (0.40, 0.67)	<0.001
Open	1.23 (1.22, 1.25)	<0.001	0.52 (0.44, 0.60)	<0.001
Other minimally invasive	1.08 (1.05, 1.11)	<0.001	0.62 (0.30, 1.28)	0.199
Percutaneous	1.39 (1.30, 1.49)	<0.001	0.74 (0.49, 1.11)	0.143
Thoracoscopic	2.15 (1.70, 2.70)	<0.001	0.82 (0.26, 2.59)	0.738
Robotic-assisted	1.64 (1.53, 1.75)	<0.001	0.52 (0.32, 0.84)	0.008
**Surgical Specialty**				
General Surgery	1.90 (1.84, 1.96)	<0.001	0.58 (0.48, 0.70)	<0.001
Gynecologic Surgery	2.08 (1.70, 2.55)	<0.001	0.50 (0.23, 1.09)	0.083
Neurosurgery	1.26 (1.19, 1.34)	<0.001	0.49 (0.27, 0.87)	0.015
Orthopedic Surgery	1.11 (1.09, 1.13)	<0.001	0.46 (0.37, 0.57)	<0.001
Otolaryngology (ENT)	2.21 (1.76, 2.77)	<0.001	0.39 (0.15, 1.05)	0.062
Plastic Surgery	2.31 (1.61, 3.32)	<0.001	0.83 (0.27, 2.56)	0.742
Thoracic Surgery	2.09 (1.71, 2.56)	<0.001	0.71 (0.23, 2.20)	0.55
Urologic Surgery	2.26 (2.06, 2.47)	<0.001	0.67 (0.50, 0.91)	0.009
Vascular Surgery	1.67 (1.56, 1.78)	<0.001	0.77 (0.53, 1.10)	0.154
**Home Support**				
Lives alone at home	1.25 (1.21, 1.29)	<0.001	0.72 (0.53, 0.98)	0.035
Lives at home with others	1.37 (1.34, 1.39)	<0.001	0.53 (0.47, 0.61)	<0.001
**Setting**				
Inpatient	1.28 (1.26, 1.29)	<0.001	0.52 (0.45, 0.60)	<0.001
Outpatient	1.52 (1.46, 1.60)	<0.001	0.66 (0.54, 0.81)	<0.001

Supplementary Table 3. Probability of postoperative transitions in independence by dementia status, operative urgency, and frailty Observed probabilities of loss of independence (LOI) among patients who were independent at baseline and gain of independence (GOI) among patients who were dependent at baseline, stratified by preoperative dementia status, operative urgency (elective vs urgent/emergent), and modified frailty index (mFI-5). LOI probabilities increase stepwise with higher frailty, emergency surgery, and dementia, whereas GOI probabilities remain low across all strata. NA indicate GOI not applicable for mFI-5 = 0 due to absence of baseline independence.

Dementia Status	Case Type	mFI-5	LOI (%)	GOI (%)
**No**	Urgent/Emergent	0	15.4	NA
		1	17.2	5.9
		2	20.5	6.9
		≥3	26.1	5.0
	Elective	0	10.1	NA
		1	10.7	7.4
		2	12.6	7.9
		≥3	16.5	6.6
**Yes**	Urgent/Emergent	0	30.7	NA
		1	32.6	2.0
		2	37.9	3.0
		≥3	42.7	2.9
	Elective	0	21.0	NA
		1	21.3	4.2
		2	24.8	4.1
		≥3	29.0	4.7
